# GAGE Cancer-Germline Antigens Are Recruited to the Nuclear Envelope by Germ Cell-Less (GCL)

**DOI:** 10.1371/journal.pone.0045819

**Published:** 2012-09-20

**Authors:** Morten F. Gjerstorff, Heike I. Rösner, Christina B. Pedersen, Katrine B. V. Greve, Steffen Schmidt, Katherine L. Wilson, Jan Mollenhauer, Hüseyin Besir, Flemming M. Poulsen, Niels Erik Møllegaard, Henrik J. Ditzel

**Affiliations:** 1 Department of Cancer and Inflammation Research, Institute for Molecular Medicine, University of Southern Denmark, Odense, Denmark; 2 Biomolecular Sciences, Department of Biology, University of Copenhagen, Copenhagen, Denmark; 3 Department of Cell Biology, The Johns Hopkins University, School of Medicine, Baltimore, Maryland, United States of America; 4 The Lundbeckfonden Center of Excellence NanoCAN, University of Southern Denmark, Odense, Denmark; 5 Protein Expression & Purification Core Facility, EMBL Heidelberg, Heidelberg, Germany; 6 Department of Cellular and Molecular Medicine, Panum Institute, University of Copenhagen, Copenhagen, Denmark; 7 Department of Oncology, Odense University Hospital, Odense, Denmark; Russian Academy of Sciences, Institute for Biological Instrumentation, Russian Federation

## Abstract

GAGE proteins are highly similar, primate-specific molecules with unique primary structure and undefined cellular roles. They are restricted to cells of the germ line in adult healthy individuals, but are broadly expressed in a wide range of cancers. In a yeast two-hybrid screen we identified the metazoan transcriptional regulator, Germ cell-less (GCL), as an interaction partner of GAGE12I. GCL directly binds LEM-domain proteins (LAP2β, emerin, MAN1) at the nuclear envelope, and we found that GAGE proteins were recruited to the nuclear envelope inner membrane by GCL. Based on yeast two-hybrid analysis and pull-down experiments of GCL polypeptides, GCL residues 209–320 (which includes the BACK domain) were deduced sufficient for association with GAGE proteins. GAGE mRNAs and GCL mRNA were demonstrated in human testis and most types of cancers, and at the protein level GAGE members and GCL were co-expressed in cancer cell lines. Structural studies of GAGE proteins revealed no distinct secondary or tertiary structure, suggesting they are intrinsically disordered. Interestingly GAGE proteins formed stable complexes with dsDNA *in vitro* at physiological concentrations, and GAGE12I bound several different dsDNA fragments, suggesting sequence-nonspecific binding. Dual association of GAGE family members with GCL at the nuclear envelope inner membrane in cells, and with dsDNA *in vitro*, implicate GAGE proteins in chromatin regulation in germ cells and cancer cells.

## Introduction

The GAGE family of highly identical, small oligomeric proteins is expressed from a locus containing 13–39 copies of nearly identical genes on the X-chromosome [1], [2]. GAGE genes are only present in higher primates and have undergone rapid expansion possibly due to strong positive selection [3]. In healthy individuals GAGE expression is limited to germ cells [4], but transcription of GAGE genes is activated upon epigenetic dysregulation in cancer cells [5]. GAGE proteins are expressed in a wide range of cancers, and their expression correlates with poor prognosis in stomach cancer, esophageal carcinoma and neuroblastoma, indicating a role in tumorigenesis [6]. GAGE proteins are known to increase cellular resistance to various cytotoxic agents by directly associating with, and affecting the level of, apoptotic regulators IRF1 and NPM1 [7], [8], but little is known about their molecular or cellular functions.

Here we report that GAGE proteins interact with GCL, a metazoan protein important for nuclear envelope integrity and germ cell development in *Drosophila* and mice [9], [10]. In both species, GCL localizes at the inner nuclear membrane, and several lines of evidence suggest that GCL inhibits transcription: GCL is required to silence transcription in *Drosophila* germ cells [11], and in mammalian cells, GCL binds the heterodimeric transcription factor DP and thereby inhibits DP-E2F-dependent genes, which are required for entry into S-phase. GCL also directly binds at least three LEM-domain proteins (emerin, MAN1 and LAP2β [12]–[14]) located at the nuclear inner membrane, and appears to require LEM-domain proteins as co-repressors *in vivo* [13], [15]. LEM-domain proteins bind lamins (nuclear intermediate filaments) and are key components of nuclear ‘lamina’ structure.

The nuclear ‘lamina’ component of the nucleoskeleton consists of networks of nuclear intermediate filaments formed by A-type or B-type lamins [16] in conjunction with barrier-to-autointegration factor (BAF) [17], [18] and LEM-domain proteins such as emerin [19], [20]. Lamins interact with chromatin and a variety of structural, regulatory and signaling proteins in the nucleus [21], and influence nuclear structure and many pathways including development, differentiation, cell proliferation and apoptosis [22], [23]. Changes in the composition or integrity of the nucleoskeleton might contribute, by mechanisms that remain poorly understood, to malignant transformation or tumor progression [24]. For example the A/T-rich-DNA-binding protein SATB1 normally forms a specialized chromatin-silencing nucleoskeletal structure only in thymocytes; breast cancer cells with high SATB1 expression show grossly misregulated gene expression [25], [26]. Cells that overexpress GCL have defective nuclear structure, implicating GCL as a structural component of the nucleus [9], [10].

We report GAGE proteins are recruited to the nuclear lamina via GCL in cells, and bind dsDNA *in vitro*. These results suggest that GAGE proteins might contribute to tumorigenesis by a mechanism that involves GCL and chromatin, and potentially also nuclear lamina organization.

## Results

### GAGE Proteins Interact with the Transcriptional Regulator Germ Cell-less (GCL)

**Figure 1:**
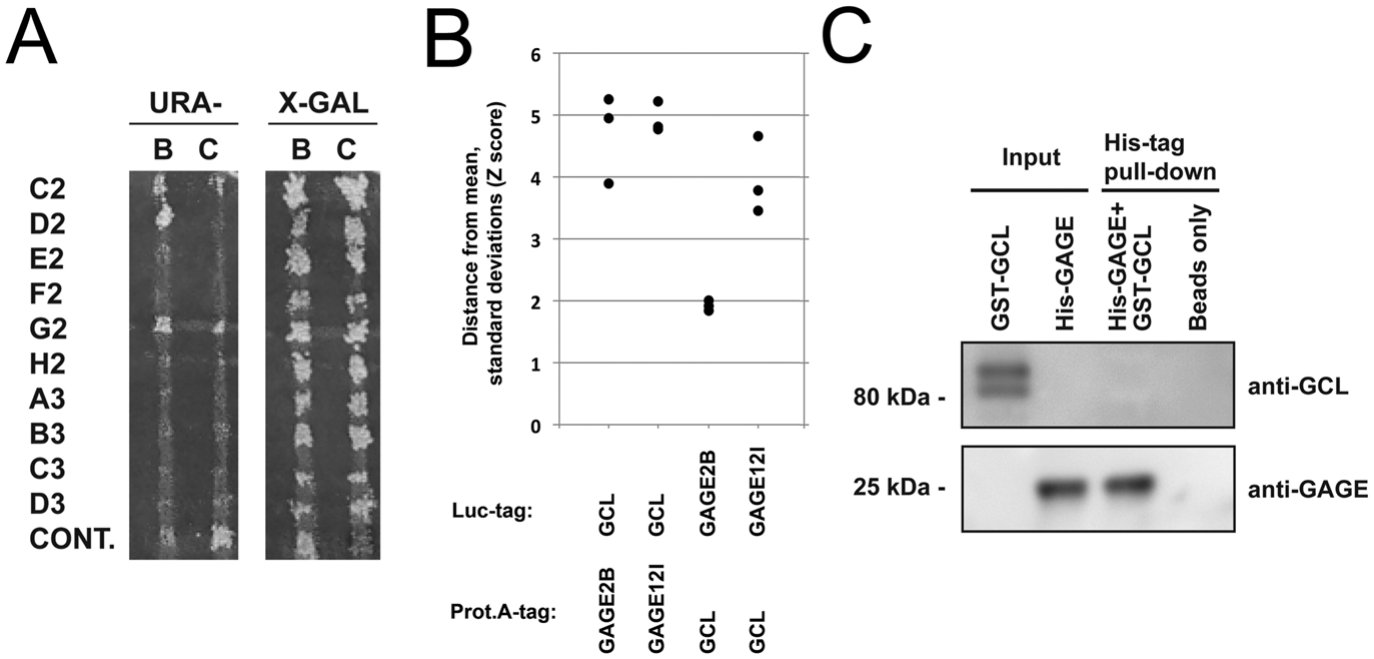
GAGE proteins interact with GCL. A. GAGE12I was used as bait in a yeast two-hybrid screen of a testis cDNA library. Plasmids encoding putative preys identified in the primary screen were rescued and retransformed into EGY42 with the bait GAGE12I vector (B) or control vector (C). Clone D2 exhibited growth on Ura- medium and blue coloration on X-Gal medium, indicating an interaction between bait and prey. The encoded prey polypeptide of D2 comprised human GCL residues 84–320, which includes the proposed BTB/POZ and BACK domains. B. Interaction between GCL and GAGE12I was verified by Lumier pull-downs from HEK293 cells co-transfected with Protein A-fused GAGE and Luciferase-fused GCL. Normalized luciferase signals (bound/input) are presented as Z scores, representing the number of standard deviations from the mean of a large set of independently derived, non-interacting Lumier protein pairs (see Materials and Methods for details). C. Pull-down assay with recombinant His-tagged GAGE12I and GST-GCL.

To better understand the cellular functions of GAGE proteins, we used a yeast two-hybrid screen to identify potential partners. TetR-based screening of a testis cDNA library using full length GAGE12I as bait yielded one clone (D2) exhibiting growth on Ura- medium and blue coloration on X-Gal medium, indicating interaction between bait and prey (Fig. 1A). The prey plasmid of D2 contained an open reading frame encoding residues 84–320 of human germ cell-less homolog 1 (GCL, alias GMCL1; NM178439.3). GCL association with GAGE12I was independently verified by Luciferase-based (Luminescence-based mammalian interactome mapping; ‘Lumier’; [27]) pull-down assays. Luciferase-tagged GCL and Protein A-tagged GAGE12I (or reciprocal contructs) were transiently expressed in HEK293 cells. We then isolated protein A fusions using IgG-coated beads and measured luciferase activity (Fig. 1B). Normalized luciferase signals (bound/input) were converted to Z scores, representing the number of standard deviations from the mean of a large set of independently derived, non-interacting Lumier protein pairs [27]. GAGE12I-GCL pairs exhibited Z scores in the range of 3.4–5.3 (Fig. 1B), clearly indicating an interaction between these proteins. In this assay GCL also associated with GAGE2B (Z scores: 1.8–5.3; Fig. 1B), which represents the GAGE2-type (GAGE2A-E) family, all of which lack a tyrosine at position 11 that can be phosphorylated in other GAGE proteins [28]. This suggested GCL associates directly or indirectly with all characterized members of the GAGE family. Since the yeast two-hybrid analysis and pull-down assays are both complex systems, we also tested potential direct binding between GAGE proteins and GCL using recombinant His-tagged GAGE12I produced in yeast and a commercially-available bacterially-expressed GCL-GST fusion protein (Fig. 1C). However, direct binding of GAGE12I and GCL was not detected under these conditions. We speculate that direct binding of GAGE and GCL might: (a) require a cofactor or posttranslational modification not provided during bacterial expression; (b) be sterically hindered by the His-tag on GAGE12I or the GST-tag on GCL. Alternatively GAGE and GCL might associate indirectly.

**Figure 2:**
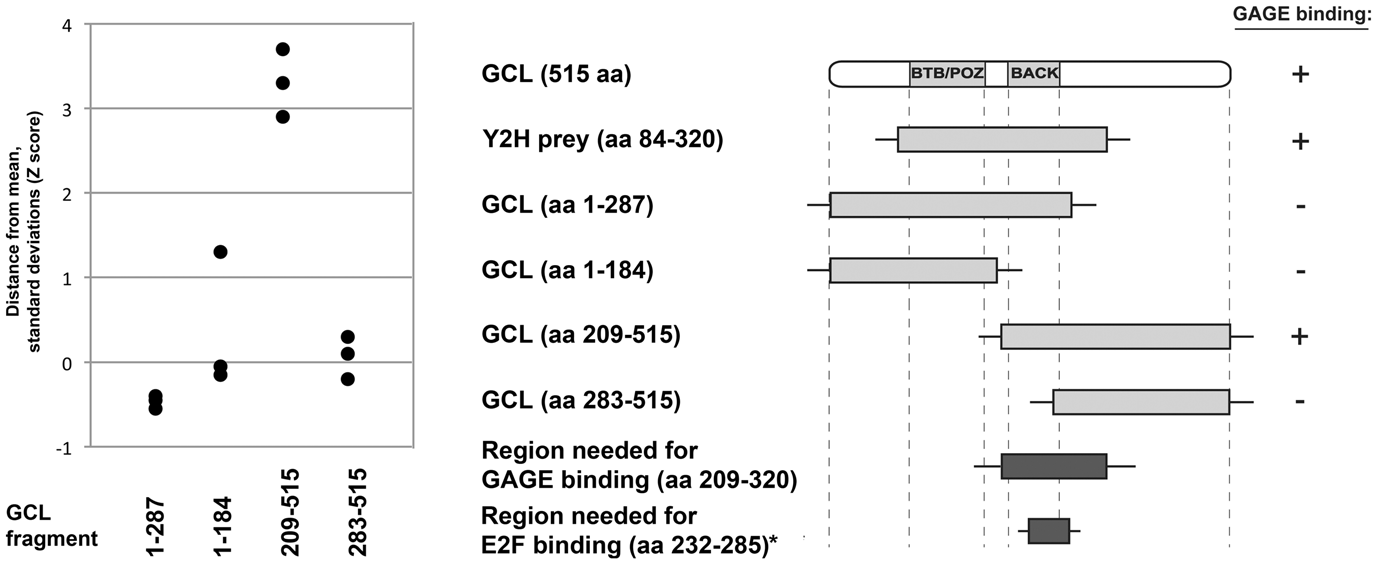
Identification of the GAGE-binding region of GCL. The binding of different GCL deletion mutants to GAGE12I was tested by Lumier assay (as in Fig. 1). The region deduced as essential for GAGE binding (GCL residues 209–320) includes residues 232–285, which are sufficient to bind transcriptional activator DP (*) [29].

Our two-hybrid screen specifically recovered GCL residues 84–320, which includes predicted BTB/POZ and BACK domains (residues 109–200 and 214–282, respectively). In other proteins, BTB/POZ domains are implicated in binding to DNA or actin [29], [30], whereas BACK-domains are mainly alpha-helical but have no generally-ascribed function [31]. To determine which GCL domains were sufficient for GAGE12I association, we repeated the Lumier assay with Protein A-tagged GAGE12I and the Luciferase-tagged GCL fragments shown in Figure 2. From these experiments, we deduced GCL residues 209–320 were both required and sufficient to associate with GAGE12I in cells. This region included the BACK domain (residues 214–282) plus 38 adjacent residues (residues 283–320). Our Jpred 3 (University of Dundee, Scotland, UK) analysis predicted that GCL residues 209–320 have several helical motifs and random coil segments (Fig. S1). Notably GCL residues 232–285 are essential to bind the DP subunit of the heterodimeric transcription factor E2F-DP [29]. This is interesting because E2F-DP-dependent genes promote proliferation (entry into S-phase) and are major targets of repression by the tumor suppressor retinoblastoma (pRb), which binds the E2F subunit [32]–[34]. GCL residues 232–285, which are essential to bind DP, are included within the deduced GAGE-association region (GCL residues 209–320) (Fig. 2 and Fig. S1). This overlap suggested at least two scenarios. First, GAGE and DP might compete for binding to GCL, and second, GAGE proteins might influence E2F-DP-dependent gene expression.

### GAGE and GCL are Co-expressed in Testis and Cancers

**Figure 3:**
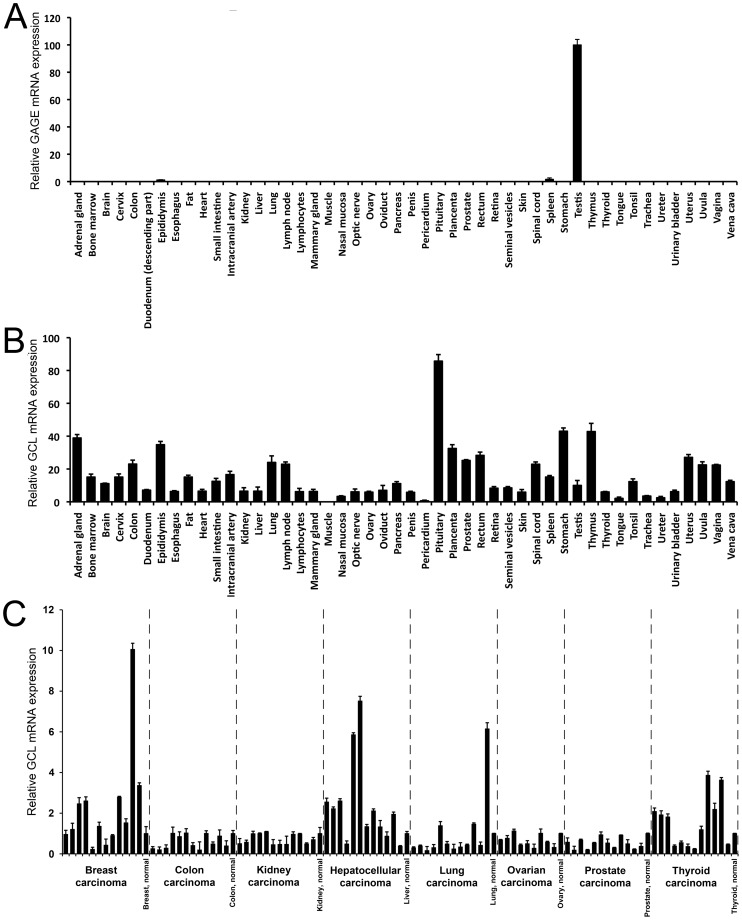
mRNA expression of GAGE and GCL genes overlaps in testis and cancers. A-C. Quantitative RT-PCR analysis of the relative expression of mRNAs encoding all known members of the GAGE family (A) or GCL (B) in normal tissues, reveals co-expression in testis. GCL mRNA was also widely expressed in different types of human cancer (C), including breast, liver, lung and thyroid cancer which were previously shown to also express GAGE proteins [4].

**Figure 4:**
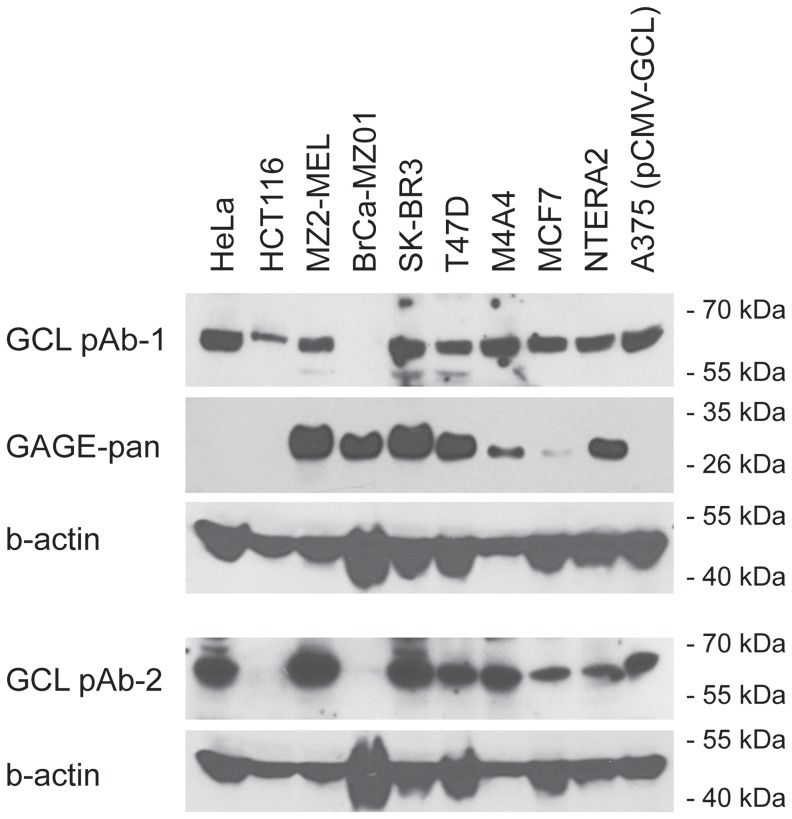
GCL and GAGE proteins are co-expressed in human cancer cells lines. GCL and GAGE protein expression was examined in 9 human cancer cells lines derived from cervix (HeLa), colon (HCT116), melanoma (MZ2-MEL), breast (BrCa-MZ01, SK-BR3, T47D, M4A4, MCF7) and embryonic cancer (NTERA2) using Western blotting. A375 melanoma cells with exogenous expression of GCL were included as positive control. Antibodies: GCL pAb1, Sigma Aldrich; GCL pAb2, clone A14, Santa Cruz Biotech; GAGE mAb, clone M3 [4], beta-actin mAb, Ab6276; Abcam.

GAGE family members are expressed detectably only in germline cells and briefly in specific cell types during primate fetal development (i.e. cells of the early ectoderm, stromal cells of the sex cord and fetal adrenal cortex cells), as determined by immunohistochemical [4], [35] and RT-PCR analysis (Fig. 3A) using antibodies and PCR primers expected to recognize all known members of the GAGE family. However, GAGE proteins are expressed in 10–40% of wide range of human cancers [4]. GCL mRNA is detected ubiquitously in Drosophila and mouse cells [10], [36]–[38], but its expression in normal and malignant human cells had not been systematically examined. To determine which human tissues might express both GAGE and GCL, we used quantitative RT-PCR to measure GAGE and GCL mRNA levels in 48 different tissues (Fig. 3A and 3B, respectively). GAGE mRNA was detected at low levels in epididymous and spleen, with high levels in testis (Fig. 3A), as expected. Consistent with mouse and Drosophila, GCL mRNA was detected in all but one normal human tissue tested; the highest levels were detected in pituitary, and the exception was muscle, where GCL mRNA was not detected (Fig. 3B). Since GCL is specifically expressed and required in Drosophila and mouse germ cells, we speculate that GCL expression in human testis is also likely localized to germ cells, where GAGE proteins are abundant [4]. GCL mRNA was also detected in all types of cancer examined, including breast, liver, lung and thyroid cancer which were previously shown to also express GAGE proteins [4], and there was no clear tendency toward up- or down-regulation of GCL mRNA expression in any particular cancer type (Fig. 3C). We next investigated the expression of GCL and GAGE proteins in a panel of human cancer cells lines of various origins using Western blotting (Fig. 4). GCL was detected in 8 out of 9 cell lines, including melanomas, breast cancers, lung cancer and embryonic carcinoma, and was co-expressed with GAGE proteins in 6 of these 8 cell lines.

Potential co-localization of GCL and GAGE proteins in sections of normal human tissues and cancers could not be examined due to the lack of suitable GCL antibodies. Since GCL mRNA is nearly ubiquitous in human tissues, one might expect the same for GCL protein. However this cannot be assumed, since GCL mRNA is repressed at the translational level in embryos as a mechanism to limit the expression of GCL protein to the germline [39]. Supporting the hypothesis that GCL protein expression is restricted, GCL-null mice phenotypes were detected in only three cell types (liver, pancreas and testis) [10]. Although GCL protein expression may be restricted in normal human tissues our analysis of human cancer cell lines suggest that GCL protein is expressed in several types of cancer.

These results were consistent with potential association of human GAGE and GCL in male germ cells and different types of cancer cells.

### GCL Recruits GAGE Proteins to the Inner Nuclear Envelope

**Figure 5:**
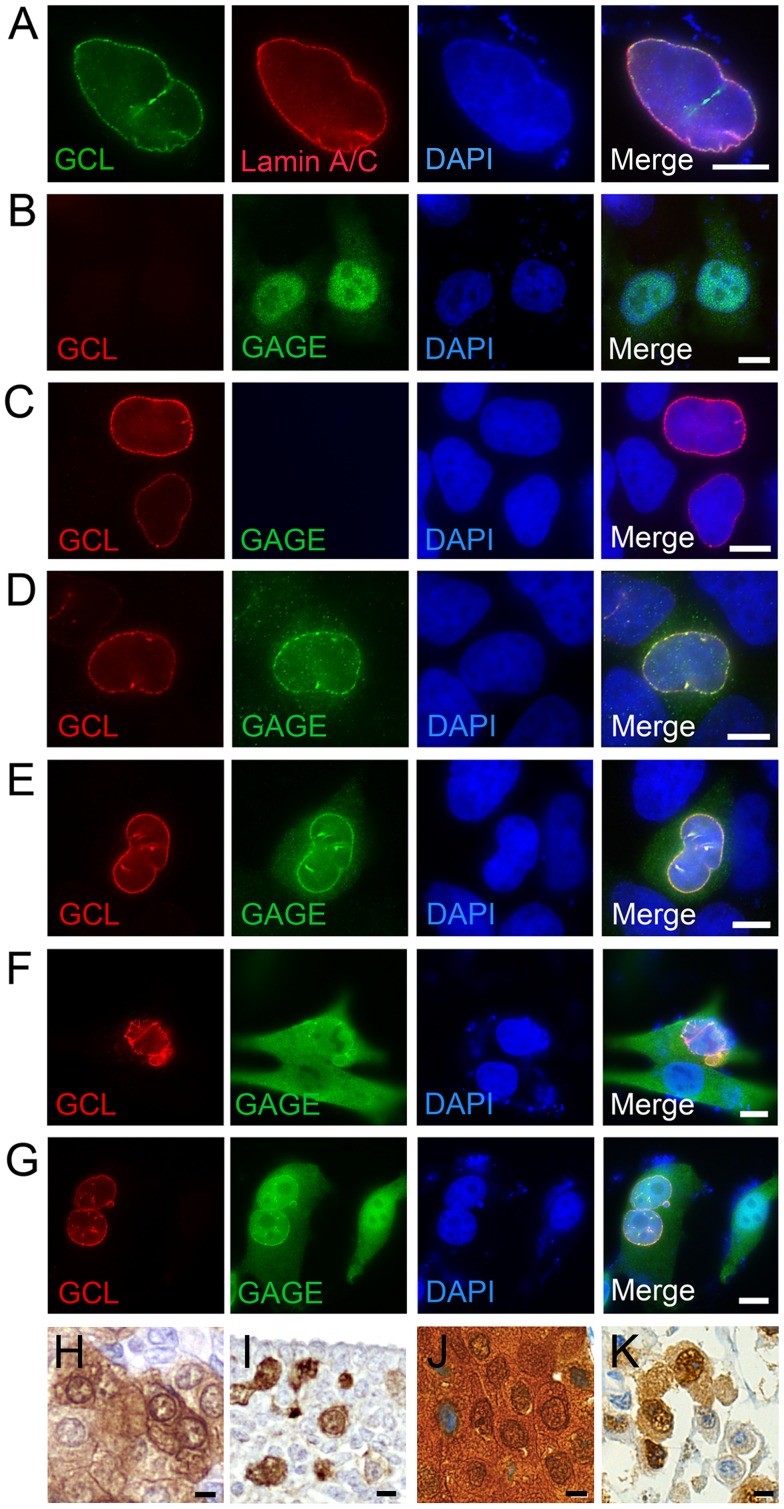
GAGE proteins are recruited to the nuclear envelope by GCL. A. Immunohistochemical double-staining of Myc-tagged human GCL and endogenous A-type lamins (lamins A/C) in transfected HeLa cells; GCL co-localized with lamins A/C at the nuclear envelope. B-E. HeLa cells were transiently transfected with GAGE12I (B), GCL-Myc (C), or GCL-Myc plus either GAGE12I (D) or GAGE1 (E). GCL localized at the nuclear envelope and recruited GAGE proteins from the nucleoplasm. F-G. Melanoma cell lines MZ2-MEL (F) and SK-MEL-31 (G), which express high levels of endogenous GAGE, were transfected to express GCL-Myc; immunostaining revealed GCL-mediated translocation of GAGE from the nucleoplasm to the nuclear envelope. H–K. Immunohistochemical analysis of endogenous GAGE proteins in human normal tissues and tumors revealed a dense GAGE signals near the nuclear envelope in cells of the fetal adrenal cortex (H), migrating primordial germ cells (I), breast carcinoma cells (J) and malignant melanoma cell (K) specimens. (GAGE = DAB/brown; Nuclei = Mayers hematoxylin/blue). Bars, 5 µm (A) and 10 µm (B-K).

GCL localizes diffusely within the nucleus and also near the nuclear envelope in *Drosophila* [38] and mouse cells [13], consistent with its direct binding *in vitro* to nuclear membrane proteins LAP2β, emerin and MAN1 [12]–[14]. To determine if GCL influenced the localization of GAGE proteins, we overexpressed Myc-tagged GCL in HeLa cells and used indirect immunofluorescence staining to verify both the nuclear localization of GCL-Myc and its co-localization with endogenous A-type lamins near the nuclear envelope (Fig. 5A). When overexpressed by itself in HeLa cells, GAGE12I localized diffusely with the brightest signals inside the nucleus (Fig. 5B), consistent with the localization of endogenous GAGE in many other cell lines and tissues [4]. Also as expected, overexpressed GCL-Myc localized both at the nuclear envelope (Fig. 5C) and diffusely in the nucleoplasm [10], [13]. Interestingly, in HeLa cells that transiently expressed both GAGE and GCL-Myc, most GAGE proteins co-localized near the nuclear envelope with GCL-Myc, as shown for GAGE12I (Fig. 5D) and GAGE1 (Fig. 5E). The same results were obtained in transfected HCT116 cells (data not shown). Similarly in two melanoma cell lines (MZ2-MEL and SK-MEL-31), transient expression of GCL-Myc shifted the distribution of endogenous GAGE proteins toward the nuclear envelope (Fig. 5F,G). We concluded exogenous GCL can recruit exogenous and endogenous GAGE proteins to the nuclear envelope.

Although association between endogenous GCL and GAGE proteins in human cells and tissues could not be investigated due to the lack of GCL antibodies suitable for immunocyto- and immunohistochemistry, GAGE proteins might have been expected to localize at the nuclear membrane in cancer cells lines demonstrated to express endogenous GCL by Western blotting (e.g. HeLa, HCT116, MZ2-MEL; Fig. 4). However, in these cells GAGE proteins were observed throughout the nuclear compartment. This could be due to a need for high cellular levels of GCL, only achievable by overexpression, to recruit enough of the nuclear GAGE proteins to expose its localization at the nuclear membrane. Thus, GAGE proteins may localize to both the nuclear membrane and nucleoplasm in cells with normal GCL levels. Notably, immunohistochemical staining of human clinical specimens with GAGE antibodies revealed non-diffuse, dense GAGE signals that appeared to concentrate near the nuclear periphery in fetal adrenal cortex cells (Fig. 5H), primordial germ cells of the mesonephros (Fig. 5I), malignant melanoma cells (Fig. 5J) and breast carcinoma cells (Fig. 5K), further substantiating that GAGE proteins associate with the inner membrane of the nuclear envelope.

### GAGE Proteins are Intrinsically Disordered

**Figure 6:**
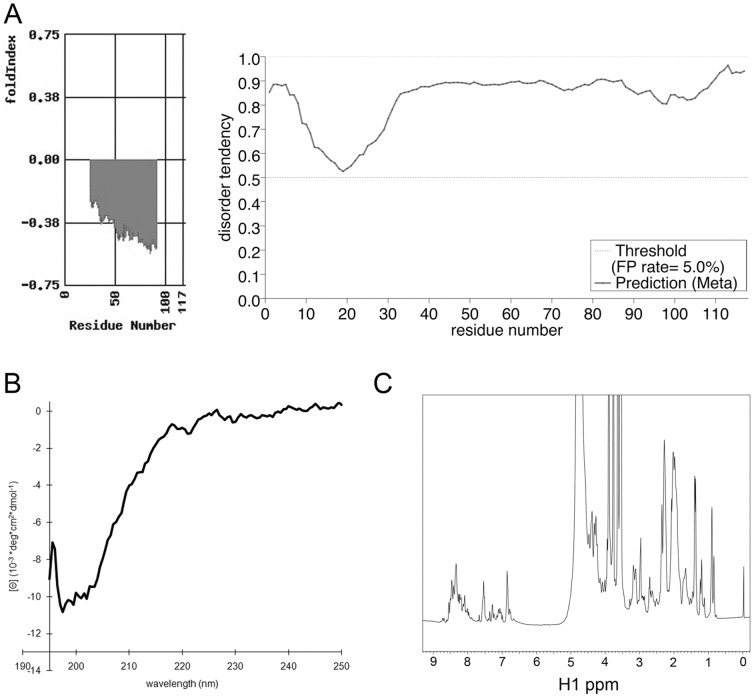
GAGE proteins are intrinsically disordered. A. Secondary structure and disorder of GAGE-12I predicted by two algorithms: FoldIndex (left panel) and metaPrDOS (right panel). B. Far-UV CD spectrum of GAGE-12I recorded from 195–250 nm of 4.5 µM GAGE-12I in 100 mM NaCl and 50 mM sodium phosphate, pH 5.5 at 25°C. The minimum at around 200 nm, plus the lack of other distinct minima, clearly indicate the protein is predominantly unfolded. C. 1D ^1^H-NMR spectrum of 4 mg/ml GAGE-12I in 100 mM NaCl, 50 mM sodium phosphate pH 5.5, 0.15 mM DSS and 10% D_2_O. The very low dispersion of the NMR signals, especially noticeable in the aliphatic region, provides a clear fingerprint of an unfolded protein.

Previous analysis of native and recombinant GAGE proteins by SDS-PAGE and size exclusion chromatography demonstrated an apparent mass of 26 kDa, which was larger than its predicted and MALDI MS-confirmed mass of ∼13 kDa [2], [4]. This anomalous migration might reflect the unusual amino acid composition of GAGE proteins, which have few hydrophobic residues (15 of 117) and many charged residues (36 of 117). However these features are also characteristic of intrinsically disordered proteins (IDPs) [40], [41]. To consider this possibility we applied two different algorithms for protein structure prediction, FoldIndex and metaPrDOS, to GAGE12I, a representative member of the GAGE family. Both algorithms predicted a high probability of disorder throughout the protein (Fig. 6A; left and right panels, respectively). Since GAGE12I is >98% identical to nearly all other known members of the GAGE family [6], this suggested that GAGE proteins generally lack secondary structure. The one exception was GAGE1; our structural prediction for GAGE1, which has a unique C-terminal domain [6], suggested this C-terminal region is alpha-helical (Fig. S1).

The potential disorder of GAGE12I was tested by circular dichroism (CD) and ^1^H Nuclear Magnetic Resonance (NMR) spectroscopy. A Far-UV CD spectrum recorded for GAGE-12I showed only one minimum at around 200 nm (Fig. 6B). This minimum, and the lack of any distinct negative ellipticity in the region from 210 to 225 nm (Fig. 6B), showed that GAGE-12I has no extended type of secondary structure. Confirming this observation, the 1D ^1^H-NMR spectrum revealed a narrow dispersion of signals (Fig. 6C). In the region of the spectrum showing signals from aliphatic sidechains, we observed no negative chemical shifts. The narrow dispersion was again clearly seen for amide proton signals in the region from approximately 7–9 ppm. We concluded GAGE12I has no distinct secondary or tertiary structure.

### GAGE Proteins Bind dsDNA at Physiological Concentrations

**Figure 7:**
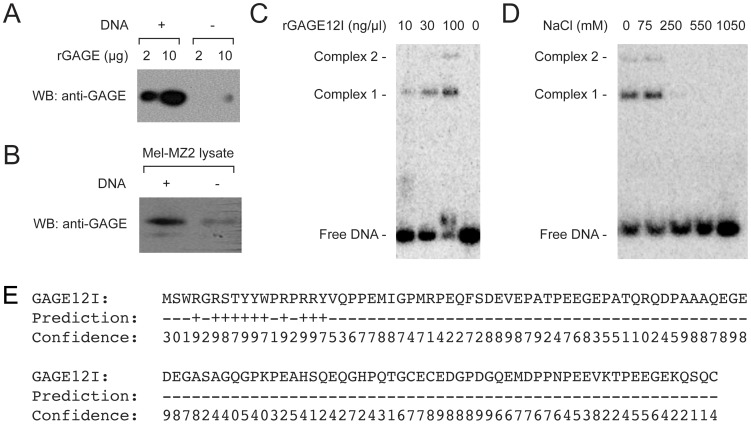
GAGE proteins bind dsDNA. A–B. Western blot of pulldown experiments testing the binding of recombinant GAGE12I (A) or endogenous GAGE proteins from MZ2-MEL lysates (B) to cellulose alone, or cellulose-immobilized native calf thymus dsDNA. C. Electrophoretic mobility shift assays. Each indicated concentration of purified GAGE12I was incubated with the ^32^P-labelled 90-bp EcoRI-PvuII DNA fragment from pUC19 at 1 pg/µl; samples were then resolved by agarose gel electrophoresis. D. Same experiment as in (C), but with addition of NaCl. E. Prediction of putative DNA binding amino acids in GAGE12I using the program BindN. +/− = binding/no binding; 0–9 = confidence of binding prediction.

GAGE proteins localize in the nucleus of both normal cells (i.e. germ cells and fetal adrenal cortex cells) and cancer cells [4]. We therefore hypothesized GAGE proteins might associate with DNA. This hypothesis was supported by pull-down experiments showing that recombinant GAGE12I (expressed in yeast and highly purified) [2]), and endogenous GAGE proteins present in MZ2-MEL melanoma cell lysate, bound to native calf thymus DNA (Fig. 7, A and B respectively).

Electrophoretic mobility shift assays were used to further analyze the direct association between GAGE proteins and dsDNA using different restriction fragments from plasmid pUC10 or pUC19 (Fig. 7C and Fig. S2). At a concentration of ∼10 ng/µl (730 nM), GAGE12I caused a subset of dsDNA molecules to migrate slowly (Fig. 7C). In the presence of 100 ng/µl GAGE12I (7.3 µM) over 50% of dsDNA migrated slowly (nucleoprotein ‘complex 1’) and we also detected an even slower-migrating complex (Fig. 7C, ‘complex 2’), suggesting potential oligomerization. GAGE12I bound several sequence-unrelated dsDNA fragments, suggesting DNA sequence-independent binding (data not shown). Both complexes were stable in 75 mM NaCl, and a small fraction of complex 1 remained stable at 250 mM NaCl (Fig. 7D).

**Figure 8:**
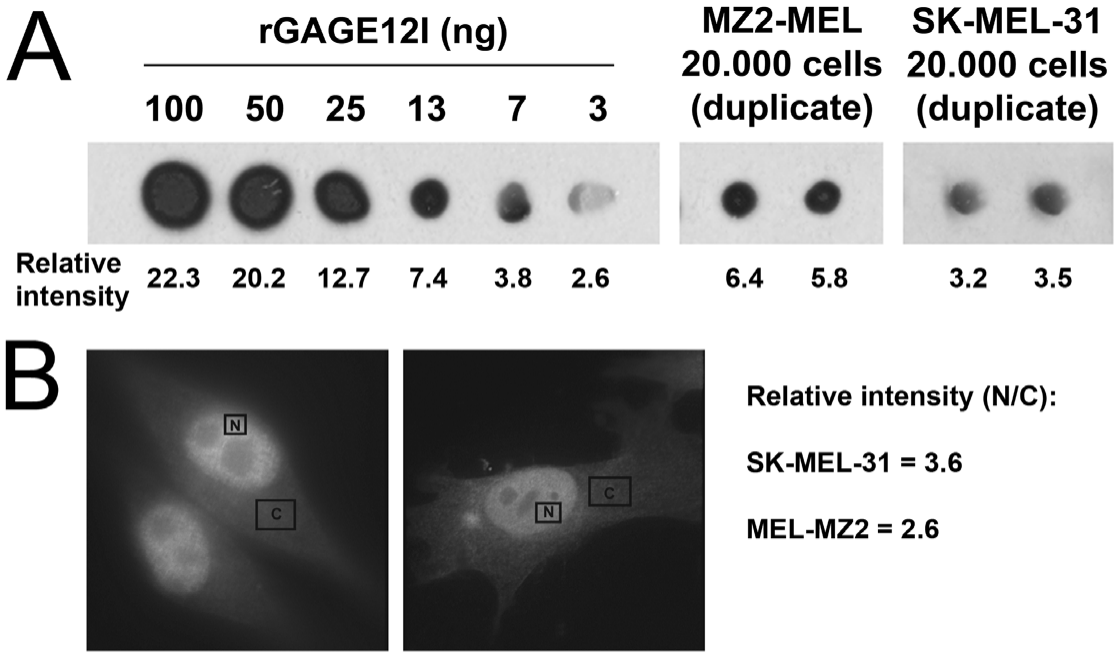
Estimated concentration of GAGE proteins in melanoma cells. A. Dot blots to estimate GAGE protein content in MZ2-MEL and SK-MEL-31 whole cell lysates by comparison to recombinant GAGE12I. Blots were probed with antibodies expected to recognize all GAGE family members; the number below each dot indicates the relative signal intensity. B. Relative quantification of GAGE proteins in nuclei and cytoplasm of MZ2-MEL and SK-MEL-31 cells based on immunostaining intensities of confocal images measured using ImageJ64 software.

We used the BindN nucleic acid prediction program [42] set to a specificity of 95% to predict potential DNA binding residues in GAGE12I. This program identified GAGE12I residues 4–17 as a putative DNA binding domain (Fig. 7E), but this needs to be verified experimentally.

The above results showed a GAGE concentration of at least 10 ng/µl (730 nM) was required to bind DNA at a concentration of 1 pg/µl (17 pM), corresponding to a molar ratio of 43∶1 (GAGE12I:DNA). To determine if these GAGE concentrations were physiologically relevant, we measured the GAGE content in MZ2-MEL and SK-MEL-31 melanoma cell lysates against known amounts of recombinant GAGE12I using dot blotting. MZ2-MEL and SK-MEL-31 cells were found to contain 0.53 pg and 0.29 pg GAGE protein per cell, respectively (Fig. 8A). Based on an estimated melanoma cell diameter of ∼15 µm (assuming a spherical form), the concentrations were conservatively calculated to be 299 ng/µl (23 µM) in MZ2-MEL cells and 164 ng/µl (12 µM) in SK-MEL-31 cells. In both cell lines, confocal microscopy suggested the GAGE signal was 2–3 times more intense in the nucleus than the cytoplasm (Fig. 8B). We concluded that the nuclear concentration of GAGE proteins in these cells is well above that (7.3 µM) required for ∼50% binding to DNA *in vitro*.

## Discussion

Functional characterization of GAGE proteins is important to understand their roles in cancer cell and germ cell biology and to evaluate their therapeutic potential as cancer targets. This study revealed that GAGE proteins interact directly or indirectly with GCL, and that GCL recruits GAGE proteins to the nuclear envelope in cells. We further demonstrated that GCL and GAGE members co-express in human testis and cancer cells. GCL is important for nuclear envelope integrity and germ cell development in both *Drosophila* and mice [9], [10]. GCL concentrates near the nuclear inner membrane, possibly reflecting its direct binding to LEM-domain proteins [12]–[14]. In *Drosophila*, GCL is required to silence transcription in germ cells [11]. In mammalian cells, GCL binds DP directly and reduces expression of E2F-DP-activated genes [29]. These findings implicate GCL as a negative regulator of DP activity. Our evidence suggests GCL also recruits GAGE proteins to the nuclear envelope. Whether this recruitment sequesters and depletes GAGE from other sites of action (as proposed for certain transcription factors; [43]), or is required for GAGE to function, or whether GCL is itself regulated by GAGE, remain unknown.

We found that GAGE proteins are intrinsically-disordered proteins that can bind dsDNA directly. Other intrinsically-disordered dsDNA-binding proteins include High Mobility Group (HMG) domain proteins and methyl-CpG binding Protein 2 (MeCP2) [44], [45]. The cancer-germline antigen PAGE4, which is both evolutionarily and structurally related to GAGE proteins (32% identity to GAGE12I), is also intrinsically disordered and exhibit DNA-binding properties [46]. Unstructured polypeptides often adopt folded structures upon binding their biological targets [41], [47]. Both the DNA-binding region of GAGE, and the structural basis of GAGE binding to DNA or chromatin, are important topics for future investigation. Our current results suggest GAGE proteins bind dsDNA in a sequence-independent manner, similar to HMG domain proteins. However potential preferential binding (e.g., to A/T-rich DNA sequences, as seen for chromatin regulator SATB1; [25]) is not ruled out. Our gel-shift experiments revealed detectable binding to 90-bp dsDNA fragments (17 pM) at a GAGE12I concentration of 0.73 µM, and approximately 50% binding at a GAGE12I concentration of 7.3 uM. These concentrations were well below the estimated GAGE protein concentrations in nuclei of melanoma cells, and suggested an *in vitro* binding stoichiometry of 43-to-1 (GAGE12I-to-DNA) at 50% binding. Since GAGE proteins form stable dimers and higher order oligomers [2], we hypothesize this high molar ratio reflects concentration-dependent oligomerization of GAGE proteins.

We do not yet understand the significance of GAGE binding to DNA *in vivo*, or whether this is influenced by GCL. However we speculate GAGE proteins might bridge chromatin to GCL-associated protein complexes at the nuclear envelope and thereby regulate chromatin organization and function [22], [48]. In the human germ cell lineage, GAGE proteins are present in primordial germ cells (PGCs) and disappear just before the first meiotic division [35], [49]. PGCs proliferate and migrate from the dorsal yolk sac via the dorsal mesentery to invade and colonize the gonadal ridges [50]. This process involves a sequence of epigenetic events that reorganize and reprogram chromatin, and ultimately shift the cell from a somatic to a germ cell state [51]. These epigenetic changes are still poorly characterized, but include genome-wide loss of DNA methylation, gradual loss of H3K9me2 and global gain of H3K27me3 [52]. Notably, epigenetic changes are also central to tumorigenesis, and phenotypic similarities between germ cells and cancer cells suggest potential activation of a gamete-related differentiation program in cancer cells [5]. We therefore speculate that GAGE family members have novel roles in higher-order chromatin reorganization and reprogramming in germ cells and cancer cells.

## Materials and Methods

### Immunoassays

Antibodies used in this study were: Mouse anti-GAGE (M3 [4]; immunocytochemistry [ICC], 1/100 dilution; western blot [WB], 1/5000 dilution), rabbit anti-Myc (A14; Santa Cruz Biotech, Heidelberg, Germany; ICC, 1/100), rabbit anti-GCL (Santa Cruz Biotech; WB, 1/1000), rabbit anti-GCL (Sigma Aldrich, Brondby, Denmark; WB, 1/1000), mouse anti-beta-Actin (Ab6276; Abcam, Cambridge, UK; WB, 1/200.000); FITC-conjugated goat anti-mouse IgG (Dako, Glostrup, Denmark; ICC, 1/400), Cy3-conjugated goat anti-rabbit IgG (Jackson ImmunoResearch Laboratories, West Grove, PA, USA; ICC, 1/400).

Western blots and cell staining were described previously [4]. For dot blots, we spotted recombinant GAGE12I or cellular lysates (made in PBS using bead mill homogenization) onto activated PVDF membrane; air-dried membranes were processed as described for Western blots and quantified using a Fusion Fx7 imager (Vilber Lourmat Deutschland GmbH, Eberhardzell, Deutschland).

### Yeast Two-hybrid Screen

The yeast two-hybrid screen was done by ProteinLinks (Pasadena, CA, USA). The GAGE12I (NM_001098418.1) coding sequence was cloned into the bait vector pCWX200 for TetR-based screening, and screened by mating the bait strain Y304 with library strain EGY42 (MATa). About 20 million independent human testes cDNA library clones (about 20 times library coverage) were screened on galactose-selective medium lacking leucine, histidine, trytophan, and uracil. All preliminary clones that grew in selective media were picked and replicated onto four selective plates to assess URA3 (or LEU2) and LacZ reporters. To further validate interactions and eliminate the possibility that spontaneous mutations caused reporter gene activation, plasmids encoding each putative partner were rescued and retransformed with GAGE12I plasmid into EGY42. Clones that activated both reporters were considered to encode GAGE12I interaction partners and subsequently sequenced.

### Luciferase Protein Interaction Assay

Gateway cloning (Invitrogen) was used to insert cDNAs encoding GAGE2B and GAGE12I, full-length GCL or GCL fragments into the pcDNA3-RLuc vector for fusion to Renilla luciferase or the pTREX-Dest30-protA vector for *Staphyloccus Aureus* protein A fusions. Proteins were transiently co-expressed in HEK293 cells using 40 ng of expression construct and 0.05 µl Lipofectamine 2000 per well in 96 well plates. After 48 hours, cells were lysed in 20 mM Tris-HCl (pH 7.5), 250 mM NaCl, 1% Triton X-100, 10 mM EDTA, 10 mM DTT, including Protease Inhibitor Cocktail (Roche, Hvidovre, Denmark), Phosphatase Inhibitor Cocktail (Roche) and Benzonase (Merck, Hellerup, Denmark). Lysates were incubated with anti-rabbit IgG-coated magnetic beads (Invitrogen) (and a sample saved to measure imput Luciferase activity) and the beads were washed 6 times. Luciferase activity was measured in both lysates and washed beads. As negative control Luciferase fusions were co-transfected with Protein A dimers. Normalized signal-to-noise ratios were calculated as (bound/input)/(bound negative control/input negative control). Z scores were then calculated from the normalized data by subtracting the population mean of a large set of independently-derived, non-interacting Lumier protein pairs and dividing by the population standard deviation (sample-population mean/population standard deviation).

### Pull-down Assay

Recombinant N-terminally His-tagged GAGE12I, purified as described [2], was bound to His-Trap Ni^2+^ beads and incubated with recombinant GST-GCL (GMCL1) (Novus Biologicals, Littleton, CO, USA) in 50 mM sodium phosphate pH 8.0, 200 mM NaCl, 0.01% Tween 20. After intensive washing proteins were eluted by boiling in SDS-PAGE sample buffer and analyzed by Western blotting.

### Cell Culture and Transfections

Melanoma cell lines MZ2-MEL were a gift from Olivier De Backer (Ludwig Institute for Cancer Research, Brussels, Belgium) [53]. HeLa, HCT116, SK-MEL-31, HEK293 and A375 cells were purchased from ATCC (Manassas, VA, USA). All cell lines were maintained in Dulbecco’s modified Eagle’s medium (Invitrogen) supplemented with 10% fetal bovine serum (FBS), penicillin (100 U/ml) and streptomycin (100 µg/ml). Serum starvation of MZ2-MEL and SK-MEL-31 cells was done in DMEM, 0.5% FBS, penicillin and streptomycin for 48 hours.

Transient DNA transfections were done using TransIT-LT1 transfection reagent (Mirus, Madison, WI, USA) per manufacturer instructions. Constructs that express GAGE-1, GAGE-2B and GAGE-12I with an N-terminal Flag-tag were described previously [4]. The pCMV6-GCL vector, used to express GCL with a C-terminal Myc-tag, was purchased from Origene (Rockville, MD, USA).

### Quantitative Analysis of GCL mRNA Expression

GCL and GAGE mRNA expression was quantified in 48 normal human tissues (Major human tissue qPCR panel; Origene) and 93 cancers of different types (Cancer survey panel, Origene). GCL expression was measured using a GCL Taqman expression assay (Hs01587063_m1; Applied Biosystems, Carlsbad, CA, USA) per manufacturer recommendations and GAGE expression was measured using a previously established assay recognizing all known GAGE members [35]. Expression levels were normalized to endogenous beta-actin levels using primers: 5′- CAA CTC CAT CAT GAA GTG TGA C -3′ and 5′- GCC ATG CCA ATC TCA TCT TG -3′.

### CD and NMR Spectroscopy

Far-UV CD measurements were performed on a Jasco J-810 spectropolarimeter. The protein concentration for all CD measurements was kept constant (4.5 µM) in a buffer consisting of 100 mM NaCl and 50 mM sodium phosphate, pH 5.5. All spectra were recorded at 298°K in a 1 mm Quarz Suprasil cuvette (Hellma, Müllheim, Germany). Four scans were accumulated. The scanning speed was 50 nm/min and the intensity was measured from 195 nm and 250 nm. All spectra were buffer corrected, and the resulting spectra were smoothed using an FFT filter as supplied by the Jasco software.

The NMR measurements were done at 298°K on a Varian INOVA 800 MHz spectrometer equipped with a cryoprobe, in aqueous buffer (100 mM NaCl, 50 mM sodium phosphate pH 5.5, 0.15 mM DSS) with 10% D_2_O, at a protein concentration of 4 mg/ml. A 1D ^1^H-NMR solvent presaturation experiment was recorded using 256 transients at a sweep width of 12000 Hz. The spectrum was referenced relative to DSS, processed and analyzed using NMRPipe [54].

### DNA Binding Assays

Recombinant GAGE12I or endogenous GAGE proteins present in lysates from MZ2-MEL cells [4], were incubated with either cellulose-immobilized native calf thymus DNA (GE Healthcare, Hilleroed, Denmark) or cellulose alone in binding buffer (20 mM Tris-HCl pH 7.4, 50 mM NaCl, 1 mM DTT, 1% BSA) for 12 hours at 4°C. Cellulose was washed 4 times in binding buffer, and bound proteins were eluted by boiling in Tris-Glycine SDS buffer, resolved by SDS-PAGE and western blotted for GAGE as described above.

For electrophoretic mobility shift assays, ^32^P-labelled dsDNA fragments (generated by restriction enzyme digest of the pUC19 derivatives pT9CT9C and pT9C [55]) were incubated with GAGE proteins in 10 µl binding buffer (20 mM Tris pH 7.4, 50 mM NaCl, 1 mM DTT) for 5 min or times indicated, at 20°C. Reactions were stopped by adding 3 µl loading buffer (50% glycerol, 100 ng/ml bromophenol blue), and resolved immediately on 6% (55∶1) polyacrylamide gels (6–8 V/cm, 23°C, 90–120 minutes). GAGE-DNA complexes were detected by autoradiography or using Phosphorimager storage screens.

## Supporting Information

Figure S1 **Protein structure predictions of GAGE1 and GCL using the Jpred3 server (University of Dundee, Scotland, UK).**
(DOC)

Figure S2 **Electrophoretic mobility shift assays of GAGE12I binding to four sequence-unrelated dsDNA fragments.**
(DOC)

## References

[1] Killen MW, Taylor TL, Stults DM, Jin W, Wang LL, et al (2011) Configuration and rearrangement of the human GAGE gene clusters. Am J Transl Res 3: 234–242. 21654878 PMC3102567

[2] Gjerstorff MF, Besir H, Larsen MR, Ditzel HJ (2010) Expression, purification and characterization of the cancer-germline antigen GAGE12I: a candidate for cancer immunotherapy. Protein Expr Purif 73: 217–222. 20546897 10.1016/j.pep.2010.05.010

[3] Liu Y, Zhu Q, Zhu N (2007) Recent duplication and positive selection of the GAGE gene family. Genetica. 10.1007/s10709-007-9179-9 17661182

[4] Gjerstorff MF, Johansen LE, Nielsen O, Kock K, Ditzel HJ (2006) Restriction of GAGE protein expression to subpopulations of cancer cells is independent of genotype and may limit the use of GAGE proteins as targets for cancer immunotherapy. Br J Cancer 94: 1864–1873. 16773077 10.1038/sj.bjc.6603163 PMC2361341

[5] Simpson AJ, Caballero OL, Jungbluth A, Chen YT, Old LJ (2005) Cancer/testis antigens, gametogenesis and cancer. Nat Rev Cancer 5: 615–625. 16034368 10.1038/nrc1669

[6] Gjerstorff MF, Ditzel HJ (2008) An overview of the GAGE cancer/testis antigen family with the inclusion of newly identified members. Tissue Antigens 71: 187–192. 18179644 10.1111/j.1399-0039.2007.00997.x

[7] Cilensek ZM, Yehiely F, Kular RK, Deiss LP (2002) A member of the GAGE family of tumor antigens is an anti-apoptotic gene that confers resistance to Fas/CD95/APO-1, Interferon-gamma, taxol and gamma-irradiation. Cancer Biol Ther 1: 380–387. 12432251

[8] Kular RK, Yehiely F, Kotlo KU, Cilensek ZM, Bedi R, et al (2009) GAGE, an antiapoptotic protein binds and modulates the expression of nucleophosmin/B23 and interferon regulatory factor 1. J Interferon Cytokine Res 29: 645–655. 19642896 10.1089/jir.2008.0099

[9] Jongens TA, Hay B, Jan LY, Jan YN (1992) The germ cell-less gene product: a posteriorly localized component necessary for germ cell development in Drosophila. Cell 70: 569–584. 1380406 10.1016/0092-8674(92)90427-e

[10] Kimura T, Ito C, Watanabe S, Takahashi T, Ikawa M, et al (2003) Mouse germ cell-less as an essential component for nuclear integrity. Mol Cell Biol 23: 1304–1315. 12556490 10.1128/MCB.23.4.1304-1315.2003 PMC141152

[11] Leatherman JL, Levin L, Boero J, Jongens TA (2002) germ cell-less acts to repress transcription during the establishment of the Drosophila germ cell lineage. Curr Biol 12: 1681–1685. 12361572 10.1016/s0960-9822(02)01182-x

[12] Mansharamani M, Wilson KL (2005) Direct binding of nuclear membrane protein MAN1 to emerin in vitro and two modes of binding to barrier-to-autointegration factor. J Biol Chem 280: 13863–13870. 15681850 10.1074/jbc.M413020200

[13] Nili E, Cojocaru GS, Kalma Y, Ginsberg D, Copeland NG, et al (2001) Nuclear membrane protein LAP2beta mediates transcriptional repression alone and together with its binding partner GCL (germ-cell-less). J Cell Sci 114: 3297–3307. 11591818 10.1242/jcs.114.18.3297

[14] Holaska JM, Lee KK, Kowalski AK, Wilson KL (2003) Transcriptional repressor germ cell-less (GCL) and barrier to autointegration factor (BAF) compete for binding to emerin in vitro. J Biol Chem 278: 6969–6975. 12493765 10.1074/jbc.M208811200

[15] Holaska JM, Wilson KL (2006) Multiple roles for emerin: implications for Emery-Dreifuss muscular dystrophy. Anat Rec A Discov Mol Cell Evol Biol 288: 676–680. 16761279 10.1002/ar.a.20334 PMC2559942

[16] Dittmer TA, Misteli T (2011) The lamin protein family. Genome Biol 12: 222. 21639948 10.1186/gb-2011-12-5-222 PMC3219962

[17] Montes de Oca R, Andreassen PR, Wilson KL (2011) Barrier-to-autointegration factor influences specific histone modifications. Nucleus 2. 10.4161/nucl.2.6.17960 PMC3324346 22127260

[18] Margalit A, Brachner A, Gotzmann J, Foisner R, Gruenbaum Y (2007) Barrier-to-autointegration factor–a BAFfling little protein. Trends Cell Biol 17: 202–208. 17320395 10.1016/j.tcb.2007.02.004

[19] Wilson KL, Berk JM (2010) The nuclear envelope at a glance. J Cell Sci 123: 1973–1978. 20519579 10.1242/jcs.019042 PMC2880010

[20] Wagner N, Krohne G (2007) LEM-Domain proteins: new insights into lamin-interacting proteins. Int Rev Cytol 261: 1–46. 17560279 10.1016/S0074-7696(07)61001-8

[21] Zastrow MS, Vlcek S, Wilson KL (2004) Proteins that bind A-type lamins: integrating isolated clues. J Cell Sci 117: 979–987. 14996929 10.1242/jcs.01102

[22] Kind J, van Steensel B (2010) Genome-nuclear lamina interactions and gene regulation. Curr Opin Cell Biol 22: 320–325. 20444586 10.1016/j.ceb.2010.04.002

[23] Prokocimer M, Davidovich M, Nissim-Rafinia M, Wiesel-Motiuk N, Bar DZ, et al (2009) Nuclear lamins: key regulators of nuclear structure and activities. J Cell Mol Med 13: 1059–1085. 19210577 10.1111/j.1582-4934.2008.00676.x PMC4496104

[24] Prokocimer M, Margalit A, Gruenbaum Y (2006) The nuclear lamina and its proposed roles in tumorigenesis: projection on the hematologic malignancies and future targeted therapy. J Struct Biol 155: 351–360. 16697219 10.1016/j.jsb.2006.02.016

[25] Han HJ, Russo J, Kohwi Y, Kohwi-Shigematsu T (2008) SATB1 reprogrammes gene expression to promote breast tumour growth and metastasis. Nature 452: 187–193. 18337816 10.1038/nature06781

[26] Simon DN, Wilson KL (2011) The nucleoskeleton as a genome-associated dynamic ‘network of networks’. Nat Rev Mol Cell Biol 12: 695–708. 21971041 10.1038/nrm3207

[27] Barrios-Rodiles M, Brown KR, Ozdamar B, Bose R, Liu Z, et al (2005) High-throughput mapping of a dynamic signaling network in mammalian cells. Science 307: 1621–1625. 15761153 10.1126/science.1105776

[28] Artemenko KA, Bergstrom Lind S, Elfineh L, Mayrhofer C, Zubarev RA, et al (2011) Optimization of immunoaffinity enrichment and detection: toward a comprehensive characterization of the phosphotyrosine proteome of K562 cells by liquid chromatography-mass spectrometry. Analyst 136: 1971–1978. 21403953 10.1039/c0an00649a

[29] de la Luna S, Allen KE, Mason SL, La Thangue NB (1999) Integration of a growth-suppressing BTB/POZ domain protein with the DP component of the E2F transcription factor. EMBO J 18: 212–228. 9878064 10.1093/emboj/18.1.212 PMC1171116

[30] Albagli O, Dhordain P, Deweindt C, Lecocq G, Leprince D (1995) The BTB/POZ domain: a new protein-protein interaction motif common to DNA- and actin-binding proteins. Cell Growth Differ 6: 1193–1198. 8519696

[31] Stogios PJ, Prive GG (2004) The BACK domain in BTB-kelch proteins. Trends Biochem Sci 29: 634–637. 15544948 10.1016/j.tibs.2004.10.003

[32] Magae J, Wu CL, Illenye S, Harlow E, Heintz NH (1996) Nuclear localization of DP and E2F transcription factors by heterodimeric partners and retinoblastoma protein family members. J Cell Sci 109 (Pt 7): 1717–1726. 10.1242/jcs.109.7.1717 8832394

[33] Wu CL, Zukerberg LR, Ngwu C, Harlow E, Lees JA (1995) In vivo association of E2F and DP family proteins. Mol Cell Biol 15: 2536–2546. 7739537 10.1128/mcb.15.5.2536 PMC230484

[34] Verona R, Moberg K, Estes S, Starz M, Vernon JP, et al (1997) E2F activity is regulated by cell cycle-dependent changes in subcellular localization. Mol Cell Biol 17: 7268–7282. 9372959 10.1128/mcb.17.12.7268 PMC232584

[35] Gjerstorff MF, Harkness L, Kassem M, Frandsen U, Nielsen O, et al (2008) Distinct GAGE and MAGE-A expression during early human development indicate specific roles in lineage differentiation. Hum Reprod 23: 2194–2201. 18611917 10.1093/humrep/den262

[36] Robertson SE, Dockendorff TC, Leatherman JL, Faulkner DL, Jongens TA (1999) germ cell-less is required only during the establishment of the germ cell lineage of Drosophila and has activities which are dependent and independent of its localization to the nuclear envelope. Dev Biol 215: 288–297. 10545238 10.1006/dbio.1999.9453

[37] Kimura T, Yomogida K, Iwai N, Kato Y, Nakano T (1999) Molecular cloning and genomic organization of mouse homologue of Drosophila germ cell-less and its expression in germ lineage cells. Biochem Biophys Res Commun 262: 223–230. 10448096 10.1006/bbrc.1999.1160

[38] Jongens TA, Ackerman LD, Swedlow JR, Jan LY, Jan YN (1994) Germ cell-less encodes a cell type-specific nuclear pore-associated protein and functions early in the germ-cell specification pathway of Drosophila. Genes Dev 8: 2123–2136. 7958883 10.1101/gad.8.18.2123

[39] Moore J, Han H, Lasko P (2009) Bruno negatively regulates germ cell-less expression in a BRE-independent manner. Mech Dev 126: 503–516. 19393317 10.1016/j.mod.2009.04.002

[40] Romero P, Obradovic Z, Li X, Garner EC, Brown CJ, et al (2001) Sequence complexity of disordered protein. Proteins 42: 38–48. 11093259 10.1002/1097-0134(20010101)42:1<38::aid-prot50>3.0.co;2-3

[41] Uversky VN, Gillespie JR, Fink AL (2000) Why are “natively unfolded” proteins unstructured under physiologic conditions? Proteins 41: 415–427. 11025552 10.1002/1097-0134(20001115)41:3<415::aid-prot130>3.0.co;2-7

[42] Wang L, Brown SJ (2006) BindN: a web-based tool for efficient prediction of DNA and RNA binding sites in amino acid sequences. Nucleic Acids Res 34: W243–248. 16845003 10.1093/nar/gkl298 PMC1538853

[43] Heessen S, Fornerod M (2007) The inner nuclear envelope as a transcription factor resting place. EMBO Rep 8: 914–919. 17906672 10.1038/sj.embor.7401075 PMC2002563

[44] Dunker AK, Cortese MS, Romero P, Iakoucheva LM, Uversky VN (2005) Flexible nets. The roles of intrinsic disorder in protein interaction networks. FEBS J 272: 5129–5148. 16218947 10.1111/j.1742-4658.2005.04948.x

[45] Adams VH, McBryant SJ, Wade PA, Woodcock CL, Hansen JC (2007) Intrinsic disorder and autonomous domain function in the multifunctional nuclear protein, MeCP2. J Biol Chem 282: 15057–15064. 17371874 10.1074/jbc.M700855200

[46] Zeng Y, He Y, Yang F, Mooney SM, Getzenberg RH, et al (2011) The cancer/testis antigen prostate-associated gene 4 (PAGE4) is a highly intrinsically disordered protein. J Biol Chem 286: 13985–13994. 21357425 10.1074/jbc.M110.210765 PMC3077599

[47] Dyson HJ, Wright PE (2005) Intrinsically unstructured proteins and their functions. Nat Rev Mol Cell Biol 6: 197–208. 15738986 10.1038/nrm1589

[48] Shaklai S, Amariglio N, Rechavi G, Simon AJ (2007) Gene silencing at the nuclear periphery. FEBS J 274: 1383–1392. 17489096 10.1111/j.1742-4658.2007.05697.x

[49] Gjerstorff MF, Kock K, Nielsen O, Ditzel HJ (2007) MAGE-A1, GAGE and NY-ESO-1 cancer/testis antigen expression during human gonadal development. Hum Reprod 22: 953–960. 17208940 10.1093/humrep/del494

[50] Burger H, de Kretser DM (1981) The Testis, 2nd ed. New York: Raven Press.

[51] Sasaki H, Matsui Y (2008) Epigenetic events in mammalian germ-cell development: reprogramming and beyond. Nat Rev Genet 9: 129–140. 18197165 10.1038/nrg2295

[52] Kota SK, Feil R (2010) Epigenetic transitions in germ cell development and meiosis. Dev Cell 19: 675–686. 21074718 10.1016/j.devcel.2010.10.009

[53] van der Bruggen P, Traversari C, Chomez P, Lurquin C, De Plaen E, et al (1991) A gene encoding an antigen recognized by cytolytic T lymphocytes on a human melanoma. Science 254: 1643–1647. 1840703 10.1126/science.1840703

[54] Delaglio F, Grzesiek S, Vuister GW, Zhu G, Pfeifer J, et al (1995) NMRPipe: a multidimensional spectral processing system based on UNIX pipes. J Biomol NMR 6: 277–293. 8520220 10.1007/BF00197809

[55] Demidov V, Frank-Kamenetskii MD, Egholm M, Buchardt O, Nielsen PE (1993) Sequence selective double strand DNA cleavage by peptide nucleic acid (PNA) targeting using nuclease S1. Nucleic Acids Res 21: 2103–2107. 8502550 10.1093/nar/21.9.2103 PMC309471

