# Supplementary material for: GAGE Cancer-Germline Antigens Are Recruited to the Nuclear Envelope by Germ Cell-Less (GCL)
Source: PLoS One. 2012 Sep 20;7(9):e45819. doi: 10.1371/journal.pone.0045819 (PMC3447759; doi:10.1371/journal.pone.0045819)
Supplement: Figure S1 — Protein structure predictions of GAGE1 and GCL using the Jpred3 server (University of Dundee, Scotland, UK). (DOC) [file pone.0045819.s001.doc]

**Figure S1:**

A. Protein structure prediction of GAGE1 using the Jpred3 server (University of Dundee, Scotland, UK).

H = Alpha helix; E=Beta-sheets; - = Random coils; U = Region different from other GAGE proteins

1-75

MSWRGRSTYYWPRPRRYVQPPEMIGPMRPEQFSDEVEPATPEEGEPATQRQDPAAAQEGEDEGASAGQGPKPEAD -----EE-E---------------------------------------------------------------HHH

76-139

SQEQGHPQTGCECEDGPDGQEMDPPNPEEVKTPEEEMRSHYVAQTGILWLLMNNCFLNLSPRKP

HHHHH-------------------------------HHHHHHHHHHHHHHHHHHHHH-------

UUUUUUUUUUUUUUUUUUUUUUUUUUUUU

B. Protein structure prediction of GCL (GMCL1) using the Jpred3 server (University of Dundee, Scotland, UK).

H = Alpha helix; E=Beta-sheets; - = Random coils; G = ”Sufficient region” for GAGE binding; D = ”Sufficient region for E2F-DP binding

1-75

MGSLSSRVLRQPRPALAQQAQGARAGGSARRPDTGDDAAGHGFCYCAGSHKRKRSSGSFCYCHPDSETDEDEEEG

---HHHHHH------------------------------------------------------------------

76-150

DEQQRLLNTPRRKKLKSTSKYIYQTLFLNGENSDIKICALGEEWSLHKIYLCQSGYFSSMFSGSWKESSMNIIEL--------------HHHHHHHHHHHHHH-----EEEEEE--EEEEEEEEEE-----HHHHH-----------EEE

151-225

EIPDQNIDVEALQVAFGSLYRDDVLIKPSRVVAILAAACLLQLDGLIQQCGETMKETVNVKTVCGYYTSAGTYGL

E-------HHHHHHHHHHH-----------HHHHHHHHH-----HHHHHHHHHHH-------HHHHHHHHHH---

GGGGGGGGGGGGGGGG

226-300

DSVKKKCLEWLLNNLMTHQNVELFKELSINVMKQLIGSSNLFVMQVEMDIYTALKKWMFLQLVPSWNGSLKQLLT

-HHHHHHHHHHHHHHHH-----------HHHHHHHHH---------HHHHHHHHHHHHHH----HHHHHHHHHHH

GGGGGGGGGGGGGGGGGGGGGGGGGGGGGGGGGGGGGGGGGGGGGGGGGGGGGGGGGGGGGGGGGGGGGGGGGGG

DDDDDDDDDDDDDDDDDDDDDDDDDDDDDDDDDDDDDDDDDDDDDDDDDDDDDD

301-375

ETDVWFSKQRKDFEGMAFLETEQGKPFVSVFRHLRLQYIISDLASARIIEQDAVVPSEWLSSVYKQQWFAMLRAE

------------HHH-----------HHHHHHHHHH----------------------EEEE-------EEEE--

GGGGGGGGGGGGGGGGGGGG

376-450

QDSEVGPQEINKEELEGNSMRCGRKLAKDGEYCWRWTGFNFGFDLLVTYTNRYIIFKRNTLNQPCSGSVSLQPRR

-----------HHHHHHH-HEEEEEE-----EEEEEEE----EEEEEEE---EEEEEE-----------EE—--E

451-515

SIAFRLRLASFDSSGKLICSRTTGYQILTLEKDQEQVVMNLDSRLLIFPLYICCNFLYISPEKKN

EEEEEEEEEEE-----EEEE-----EEEEE-----EEEEEE-----E-HHHHHHEEEEEE-----
