# Supplementary material for: GAGE Cancer-Germline Antigens Are Recruited to the Nuclear Envelope by Germ Cell-Less (GCL)
Source: PLoS One. 2012 Sep 20;7(9):e45819. doi: 10.1371/journal.pone.0045819 (PMC3447759; doi:10.1371/journal.pone.0045819)
Supplement: Figure S2 — Electrophoretic mobility shift assays of GAGE12I binding to four sequence-unrelated dsDNA fragments. (DOC) [file pone.0045819.s002.doc]

**Figure S2:** Electrophoretic mobility shift assays of GAGE12I binding to four sequence-unrelated dsDNA fragments. Purified GAGE12I was incubated with 1 pg/µl of different ^32^P-labelled dsDNA restriction fragments of a modified pUC10 vector (303, 300, 212, 209 bp). GAGE12I concentrations were 0 ng/µl (lane 1, 4, 7, 10), 50 ng/µl (lane 2, 5, 8, 11) or 100 ng/µl (lane 3, 6, 9, 12). Samples were then resolved by agarose gel electrophoresis and detected by using Phosphorimager storage screens.

**1 2 3 4 5 6 7 8 9 10 11 12**


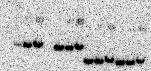


303 bp 300 bp 212 bp 209 bp
